# Gastric submucosal abscess caused by *Edwardsiella tarda* infection: a case report

**DOI:** 10.1186/s12876-020-01446-1

**Published:** 2020-09-14

**Authors:** Koshi Ota, Hiroki Yamanoue, Nobuyuki Aizawa, Naoyuki Suzuki, Kanna Ota, Akira Takasu

**Affiliations:** 1 Department of Emergency Medicine, Osaka Medical College, 2-7 Daigaku-machi, Takatsuki City, Osaka 596-8686 Japan; 2 Shizuoka Tokushukai Hospital, Shizuoka, Japan

**Keywords:** *Edwardsiella tarda*, Endoscopic ultrasound (EUS)-guided drainage, Gastric submucosal abscess, Intra-abdominal abscess

## Abstract

**Background:**

*Edwardsiella tarda* is a motile, facultatively anaerobic gram-negative bacillus that is isolated from a wide spectrum of animals in aquatic environments but rarely causes infection in humans. Here, we describe the case of a gastric submucosal abscess caused by *E. tarda* infection.

**Case presentation:**

The patient was a 74-year-old man with a history of hypertension and chronic alcohol consumption who was admitted to our hospital for abdominal pain, appetite loss and vomiting. Contrast-enhanced computed tomography (CT) revealed choledocholithiasis in the common bile duct, a gastric wall abscess and an intra-abdominal abscess. Endoscopic ultrasound (EUS)-guided drainage with antibiotics successfully cured the patient.

**Conclusion:**

The combination of CT, endoscopy and EUS-guided drainage with antibiotic therapy might be effective for diagnosis and treatment of a gastric submucosal abscess caused by *E. tarda* infection.

## Background

*Edwardsiella tarda* is a motile, facultatively anaerobic gram-negative bacillus that is commonly found in aquatic environments [1]. *E. tarda* is an important pathogen of fish living in both freshwater and saltwater. In human, gastroenteritis is observed in 80% of all *E. tarda* foodborne infections [2, 3]. The remaining 20% of human *E. tarda* infections are extraintestinal diseases including myonecrosis, soft tissue infections, meningitis, peritonitis with sepsis, bacteremia and wound infections [1, 4].

A gastric submucosal abscess is primarily caused by bacterial infection of the gastric wall and often has a poor prognosis [5]. This type of abscess is rare because of the rich blood supply to the gastric wall and the bactericidal action of gastric acid [6]. Treatment strategies have included endoscopic drainage and systemic antibiotics with or without surgical resection of the abscess [7].

Here, we describe a rare case in which a patient developed a gastric submucosal abscess caused by *E. tarda* infection. Endoscopic ultrasound (EUS)-guided drainage with antibiotic therapy successfully cured the patient. We obtained verbal and written informed consent from the patient for reporting this case.

## Case presentation

A 74-year-old man was admitted to our hospital with a 4-day history of abdominal pain, appetite loss and vomiting. He took 40 mg telmisartan, 5 mg amlodipine besilate combined drug and 2.5 mg amlodipine besilate for hypertension with 100 mg celecoxib, 37.5 mg tramadol hydrochloride/325 mg acetaminophen and 0.5 mg etizolam. He drank 540 mL sake (rice wine; about 75 g ethanol/540 mL sake) [8] every day. He had smoked half a pack of tobacco for 50 years and quit 5 years ago. His vital signs upon arrival at the emergency room were as follows: body temperature, 37.2 °C; pulse rate, 157 beats/min; respiratory rate, 32 breaths/min; blood pressure, 111/81 mmHg; and oxygen saturation, 100% on oxygen mask 6 L/min. His conscious level was alert. He had icterus on his conjunctiva. The neck was supple. His heart sounds were unremarkable. The lungs were clear to auscultation. His abdomen was not distended. There was no tenderness or hepatosplenomegaly. Neurological examination was unremarkable. Hematological and biochemical testing showed leukocytosis (white blood cell count of 13,900/mm^3^ with 92.3% neutrophils) and elevated C-reactive protein (26.93 mg/dL). He had no anemia (hemoglobin, 15.8 g/dL) or thrombocytopenia (323,000/μL). Other relevant data were as follows: blood urea nitrogen, 45.0 mg/dL; creatinine, 1.66 mg/dL; Na, 124 mEq/L; Cl, 92 mEq/L; and K, 5.8 mEq/L. Serum bilirubin was markedly elevated at 6.8 mg/dL (normal range: 0.3–1.3 mg/dL) with direct bilirubin elevation of 5.0 mg/dL (normal range: 0–0.3 mg/dL). Other liver enzymes were also elevated as follows: serum aspartate aminotransferase, 67 IU/L (normal range: 13–37 IU/L); serum alanine aminotransferase, 39 IU/L (normal range: 8–45 IU/L); lactate dehydrogenase, 612 IU/L (normal range: 122–228 IU/L); alkaline phosphatase, 1228 IU/L (normal range: 118–335 IU/L); and gamma-glutamyltransferase, 602 IU/L (normal range: 12–49 IU/L).

**Fig. 1:**
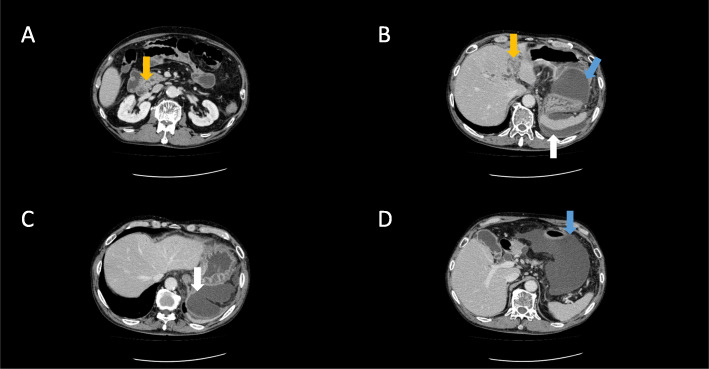
Contrast-enhanced computed tomography findings. Scans showed the following: (**a**) a dilated common bile duct with choledocholithiasis (orange arrow); (**b**) intrahepatic biliary dilatation (orange arrow), an intra-abdominal abscess surrounded by smoothly thickened and enhanced peritoneum (white arrow) and a gastric submucosal abscess (blue arrow); (**c**) an intra-abdominal abscess surrounded by smoothly thickened and enhanced peritoneum (white arrow); and (**d**) a gastric submucosal abscess (blue arrow)

Contrast-enhanced computed tomography (CECT) on the day of admission revealed choledocholithiasis in the common bile duct with intrahepatic biliary dilatation (Fig. 1a and b) and also showed a gastric wall abscess and intra-abdominal abscess around the spleen (Fig. 1c and d).

**Fig. 2:**
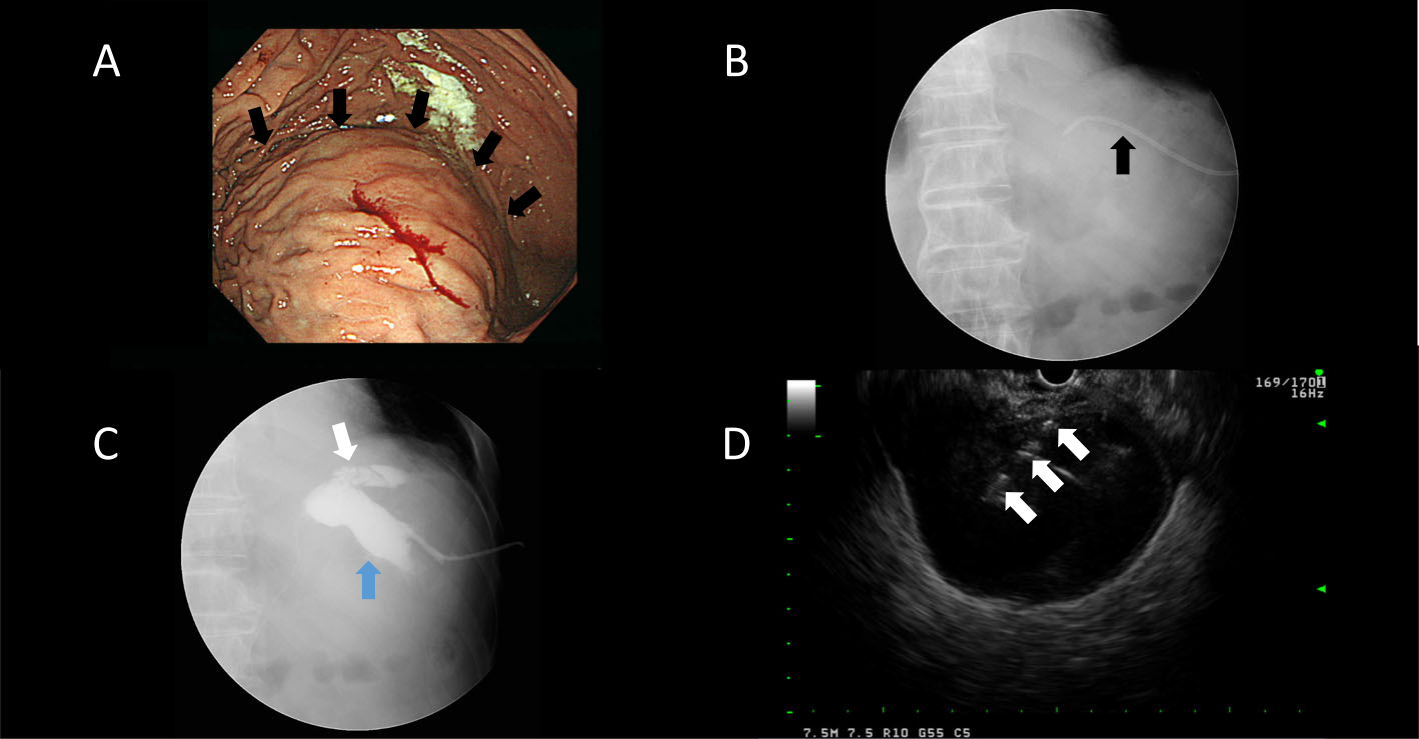
Endoscopic and endoscopic ultrasound findings. **a** An elevated lesion similar to a submucosal tumor was observed at the greater curvature of the stomach (arrows). **b** An 8 Fr catheter was placed in the intra-abdominal abscess before fistulography. **c** Fistulography showed connection between the intra-abdominal abscess (white arrow) and gastric submucosal abscess (blue arrow). Size of gastric submucosal abscess was larger than that of intra-abdominal abscess. **d** The gastric submucosal abscess was accessed under guidance using a 19-gauge fine-needle aspiration needle (arrows)

Cefoperazone sodium/sulbactam sodium was administered soon after blood culture. Endoscopic retrograde cholangiopancreatography (ERCP) was performed and 6 gallstones in the common bile duct were removed on hospital day 4. Percutaneous drainage with an 8 French (Fr) catheter through the left side of the back was performed for the intra-abdominal abscess on hospital day 7 (Fig. 2b). The abscess had a bile-like color and bilirubin was markedly elevated at 8.1 mg/dL with direct bilirubin of 4.9 mg/dL. Both blood and abscess cultures were positive for *E. tarda.* On endoscopic examination, a large mass was noted at the greater curvature of the stomach (Fig. 2a, Supporting figure 1A, B, and C, which showed the greater curvature of the stomach (arrow)). Fistulography revealed connection between the intra-abdominal abscess and gastric submucosal abscess (Fig. 2b and c). The size of the intra-abdominal abscess was decreased; however, the size of the gastric submucosal abscess was not changed.

**Fig. 3:**
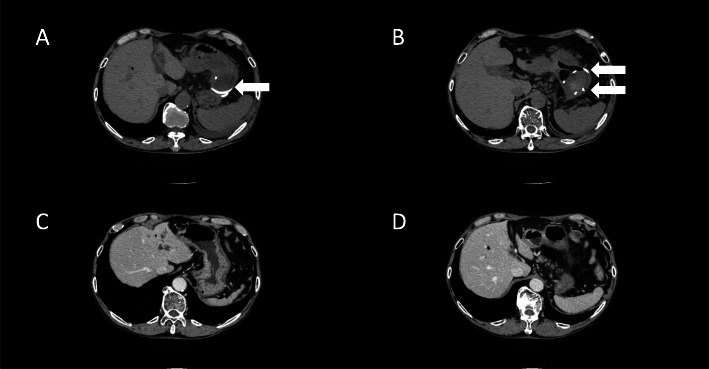
Computed tomography (CT) findings. **a** and **b** Plain CT scans taken on hospital day 26. A 5 Fr pigtail catheter was placed in the gastric submucosal abscess (arrows). **c** and **d** Contrast-enhanced CT scans taken 4 months after discharge. No gastric submucosal abscess and intra-abdominal abscess were shown

EUS-guided drainage with a 5 Fr pigtail catheter was performed for the gastric submucosal abscess on hospital day 22 (Fig. 2d, Supporting figure 1D and E, and Supporting figure 2, which showed layer of the stomach). The catheter successfully decreased the abscess size (Fig. 3a and b). Abscess culture at this time revealed *Enterococcus faecalis* and *Candida*, which were thought to be microbial substitution caused by long-term antibiotic therapy. The bilirubin level in the gastric submucosal abscess was elevated at 5.2 mg/dL with direct bilirubin of 2.4 mg/dL. The catheter was placed for 14 days then removed on hospital day 35. The patient was discharged on hospital day 36 without any complications. A CT scan taken 4 months after discharge showed complete disappearance of both the gastric submucosal abscess and intra-abdominal abscess (Fig. 3c and d).

## Discussion and conclusion

To our knowledge, this is the first case of gastric submucosal abscess caused by *E. tarda* bacteremia. Streptococci are the most commonly isolated bacteria from gastric wall abscesses (75% of cases) [5]. However, to date, *E. tarda* has never been reported to cause a gastric submucosal abscess.

*E. tarda* is a motile, facultatively anaerobic gram-negative bacillus, a member of the family Enterobacteriaceae, which is found widely in nature and commonly in aquatic environments [1]. *E. tarda* is broadly isolated from fish, amphibians, reptilians, birds and mammals. *E. tarda* rarely causes infection in humans because it is not a member of the normal human flora. Ingestion of contaminated food or water and exposure to aquatic environments were reported to be the route of *E. tarda* infection, which introduces the bacterium into the gastrointestinal tract. Our patient had no previous reported risk factors associated with *E. tarda* bacteremia except chronic alcohol consumption and hepatobiliary diseases [9].

In our case, both blood culture and ascites revealed *E. tarda*, however, culture of the gastric submucosal abscess detected *Enterococcus faecalis* and *Candida*. We consider that the difference in detected pathogens was caused by microbial substitution caused by long-term antibiotic therapy. Although the isolated *E. tarda* strain was susceptible to most antimicrobials (data not shown), cefoperazone sodium/sulbactam sodium was administered and our patient recovered. *E. tarda* bacteremia has been reported to have a high mortality rate of 40–50% [4].

A gastric submucosal abscess is a rare condition because of the bactericidal action of gastric acid and the rich blood supply to the gastric wall [6]. There are three possible infection routes: 1) direct infection to the gastric wall, 2) hematogenous infection from other infection foci and 3) lymphatic spread from other infection foci [7]. In our case, direct infection from cholangitis with choledocholithiasis might be the route, and a similar case had been previously reported in Japan [10]. Bilirubin levels were elevated in both the gastric submucosal abscess and intra-abdominal abscess, which indicated that cholangitis due to choledocholithiasis was the etiology of this case. Biliary tract is one of the causes of intra-abdominal abscess due to increased permeability of the inflamed biliary epithelium [11–13], however, we could not find the evidence of the causal relationship between cholangitis and intra-abdominal abscess because bile culture was not taken during ERCP. Streptococci are the most common bacteria isolated from gastric wall abscesses (75% of cases) [5]. *E. tarda* has never been reported to cause a gastric submucosal abscess.

Treatment for a gastric submucosal abscess includes surgical gastric resection and percutaneous drainage. Recent technical advances allow for EUS-guided drainage and seemed to be effective. The pus from the gastric submucosal abscess was too viscous for aspiration, however, a 5 Fr catheter successfully drained the pus via the nose in this case.

In conclusion, we encountered an extremely rare case of gastric submucosal abscess caused by *E. tarda* bacteremia. Both diseases are considered to be very rare and result in high mortality. The combination of CT, endoscopy and EUS-guided drainage with antibiotic therapy might be effective for diagnosis and treatment of gastric submucosal abscess caused by *E. tarda* bacteremia.

## Supplementary information

**Additional file 1.**

## Data Availability

The datasets used and/or analysed during the current study available from the corresponding author on reasonable request.

## Abbreviations

CECT: Contrast-enhanced computed tomography
CT: Computed tomography
ERCP: Endoscopic retrograde cholangiopancreatography
EUS: Endoscopic ultrasound

## References

[1] Leung YK, Siame BA, Tenkink BJ, Noort RJ, Mok Y (2012). Edwardsiella tarda e virulence mechanisms of an emerging gastroenteritis pathogen. Microbes Infect.

[2] Hirai Y, Asahata-tago S, Ainoda Y (2015). Edwardsiella tarda bacteremia. A rare but fatal water- and foodborne infection : Review of the literature and clinical cases from a single centre. Can J Infect Dis Med Microbiol.

[3] Taguchi H, Tamai T, Numata M, Maeda H (2014). Endoscopic ultrasonography-guided transmural drainage of an infected hepatic cyst due to Edwardsiella tarda: a case report. Clin J Gastroenterol.

[4] Wang I-K, Kuo H-L, Chen Y-M (2005). Extraintestinal manifestations of Edwardsiella tarda infection. Int J Clin Pr.

[5] Choong NWW, Levy MJ, Rajan E, Kolars JC (2003). Intramural gastric abscess : case history and review. Gastrointest Endosc.

[6] Kim SB, Oh MJ, Lee SH (2015). Gastric subepithelial lesion complicated with abscess : case report and literature review. World J Gastroenterol.

[7] Mandai K, Amamiya K, Uno K, Yasuda K (2016). Endoscopic ultrasonography in the diagnosis and treatment of a gastric wall abscess. J Med Ultrason.

[8] Miyazaki M, Une H (2001). Japanese alcoholic beverage and all cause mortality in Japanese adult men. J Epidemiol.

[9] Suzuki K, Yanai M, Hayashi Y, Otsuka H (2018). Edwardsiella tarda bacteremia with psoas and epidural abscess as a food-borne infection : a case report and literature review. Intern Med.

[10] Sugimoto T, Hirano T, Nakai N, Fujimoto J (2009). A case of gastric wall abscess with choledocholithiasis and cholangitis. Nihon Rinsho Geka Gakkai Zasshi.

[11] Altemeier WA, Culbertson WR, Fullen WD, Shook CD (1973). Intra-abdominal abscesses. Am J Surg.

[12] Singh S, Khardori NM (2012). Intra-abdominal and pelvic emergencies. Med Clin North Am.

[13] Zimmer V, Lammert F (2015). Acute bacterial cholangitis. Viszeralmedizin..

