# Supplementary figures and images for: Gastric submucosal abscess caused by Edwardsiella tarda infection: a case report
Source: BMC Gastroenterol. 2020 Sep 14;20:299. doi: 10.1186/s12876-020-01446-1 (PMC7491108; doi:10.1186/s12876-020-01446-1)

A

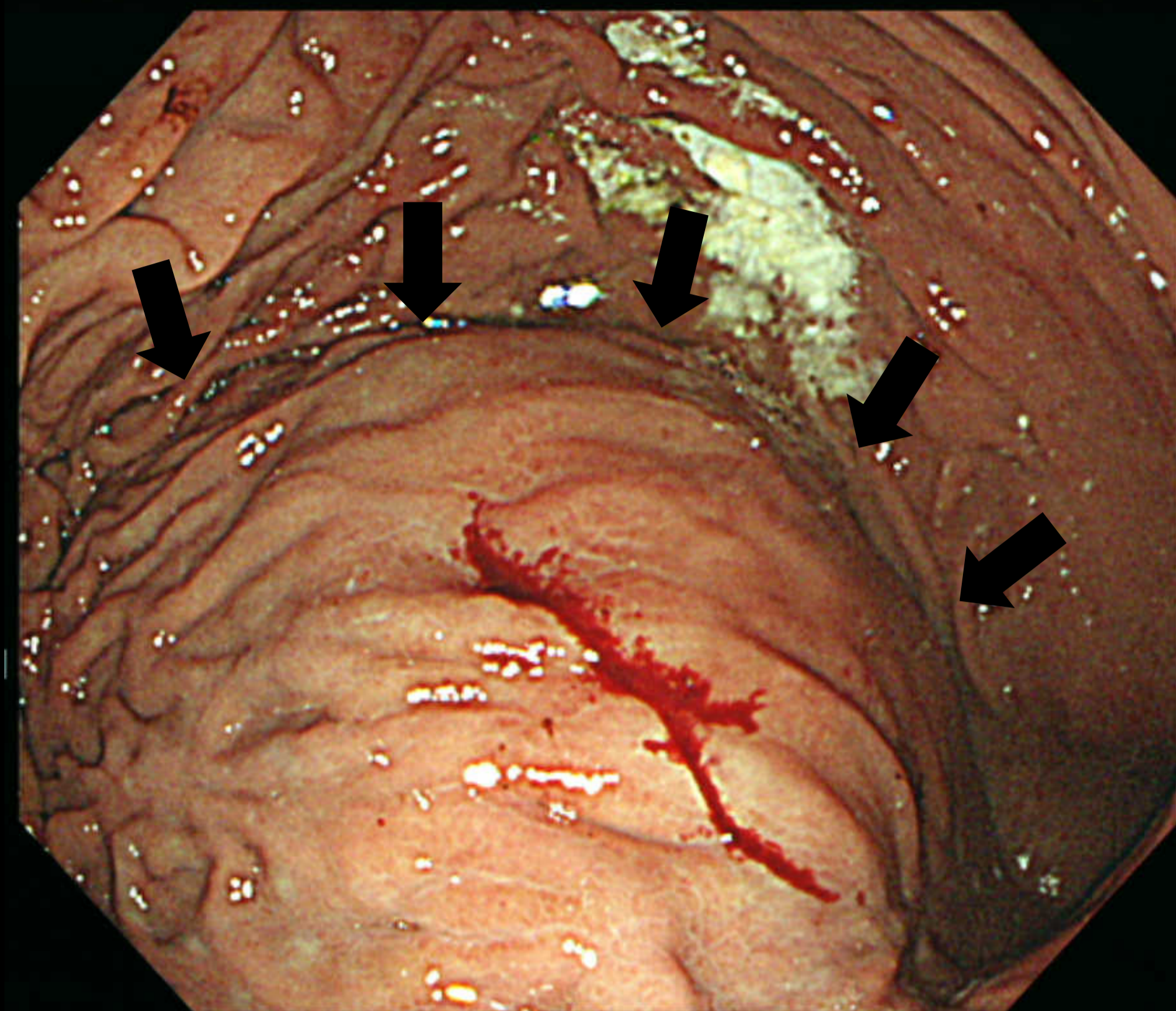

B

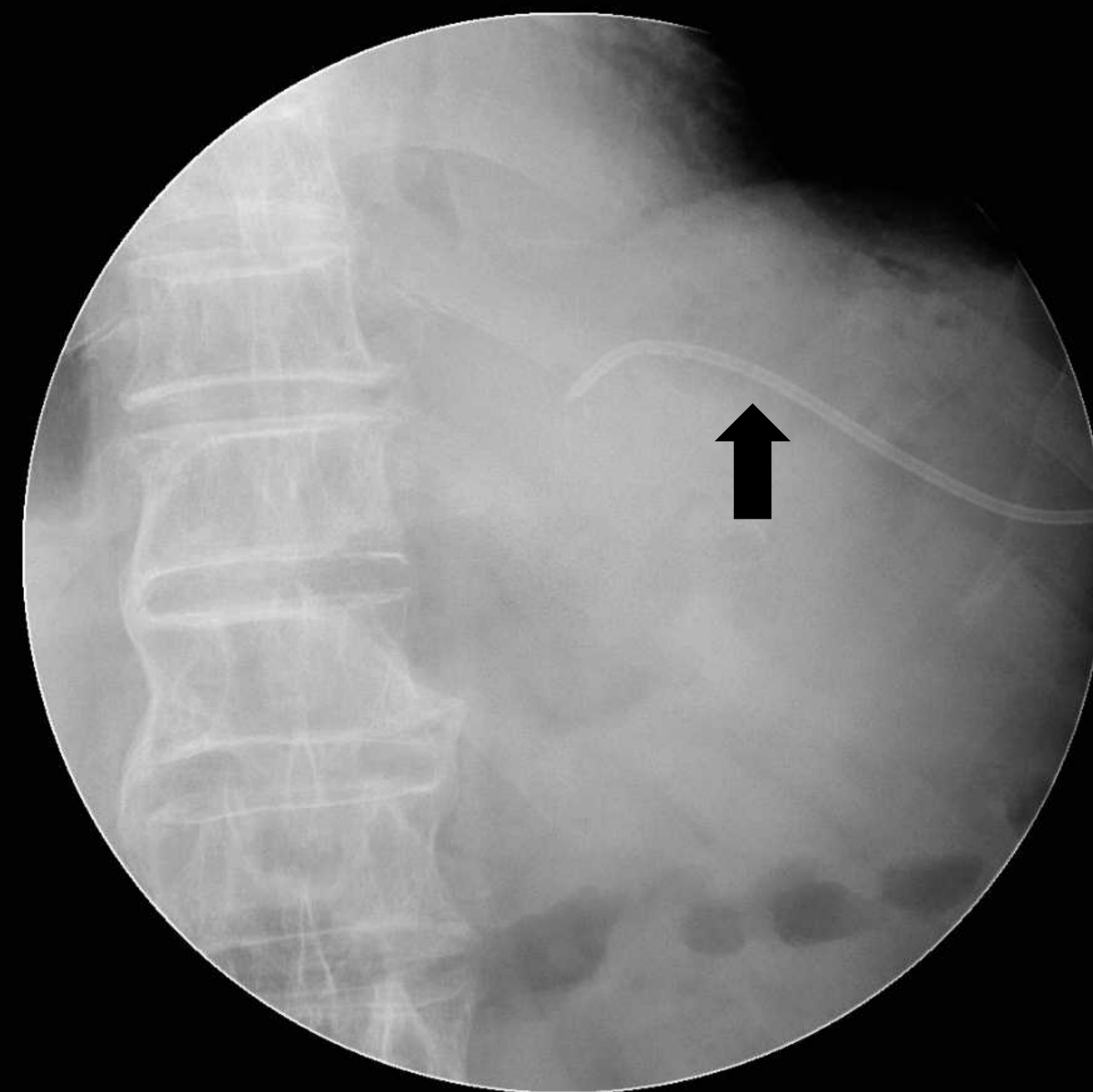

C

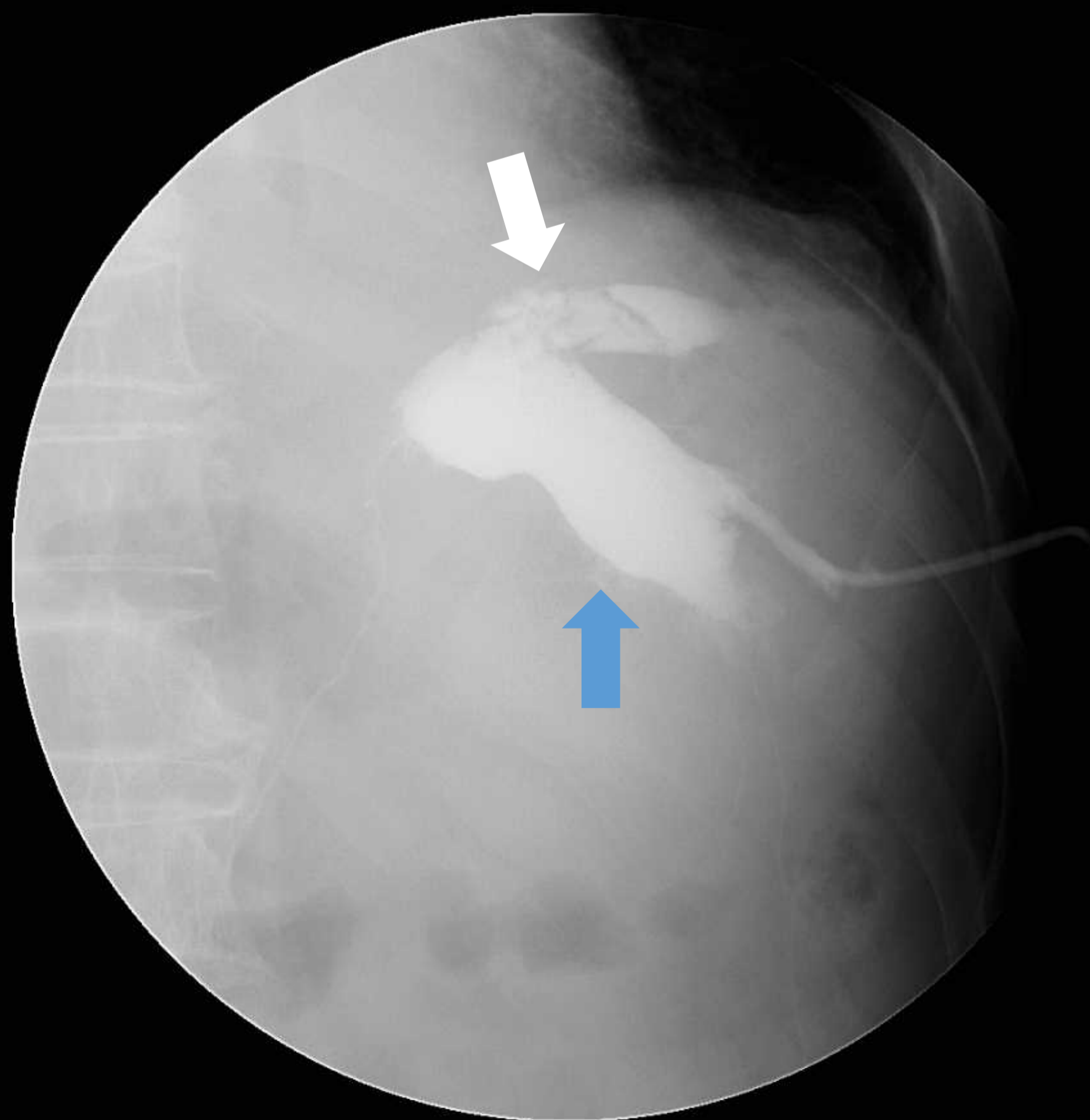

D

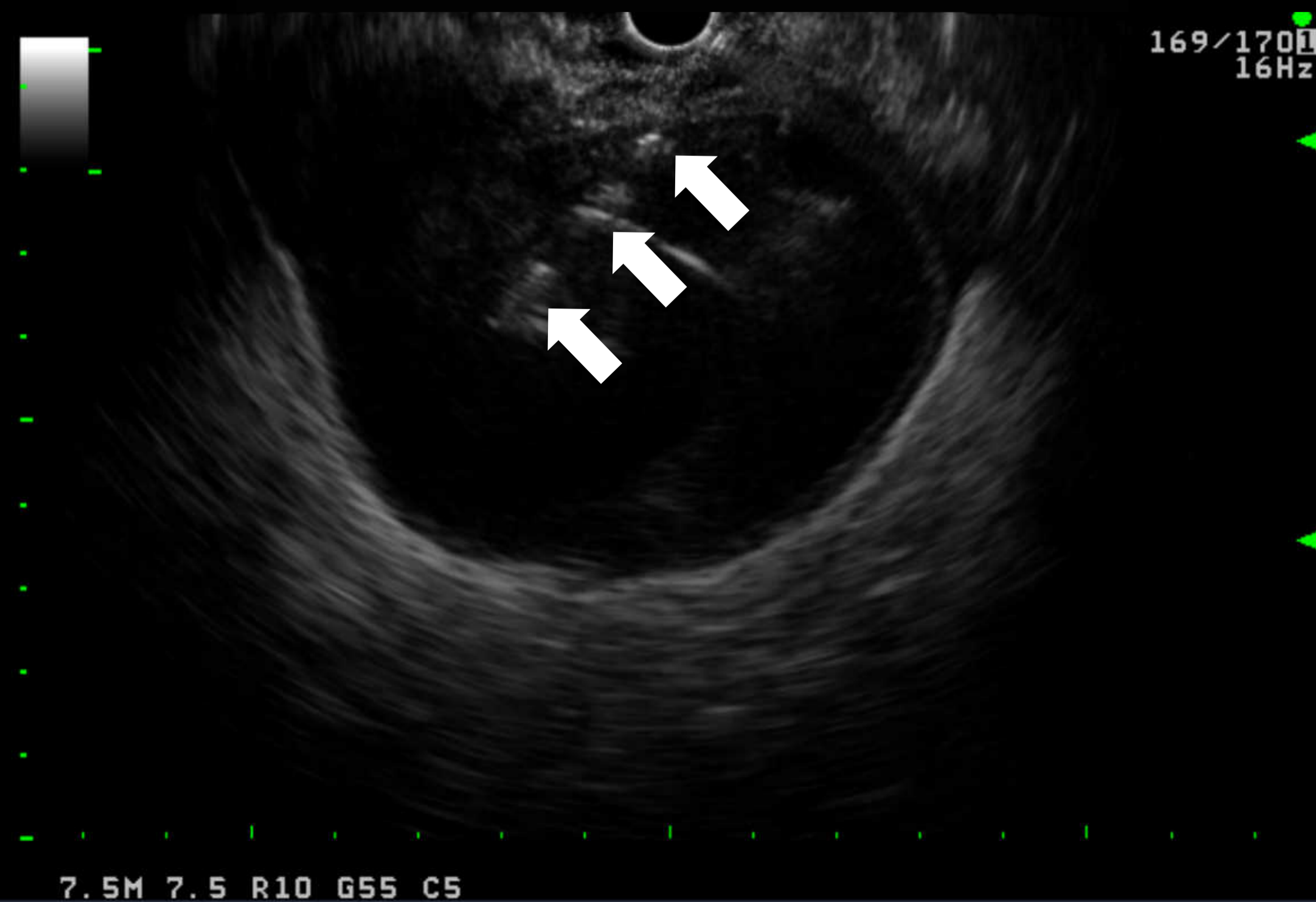

Supplement: Supplementary file 1 — Additional file 1. [file 12876_2020_1446_MOESM1_ESM.zip › Edwardsiella2newR4.pdf]

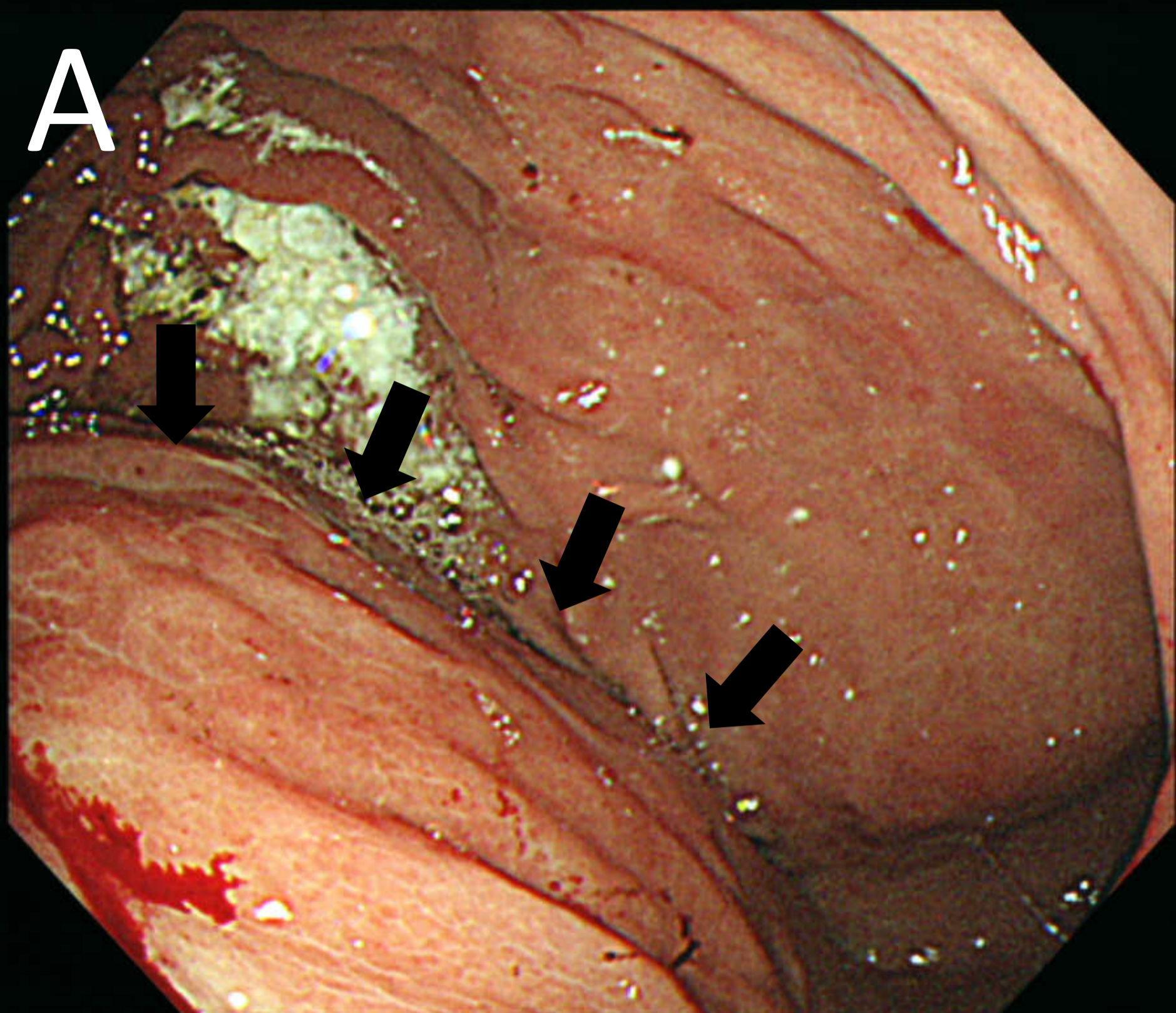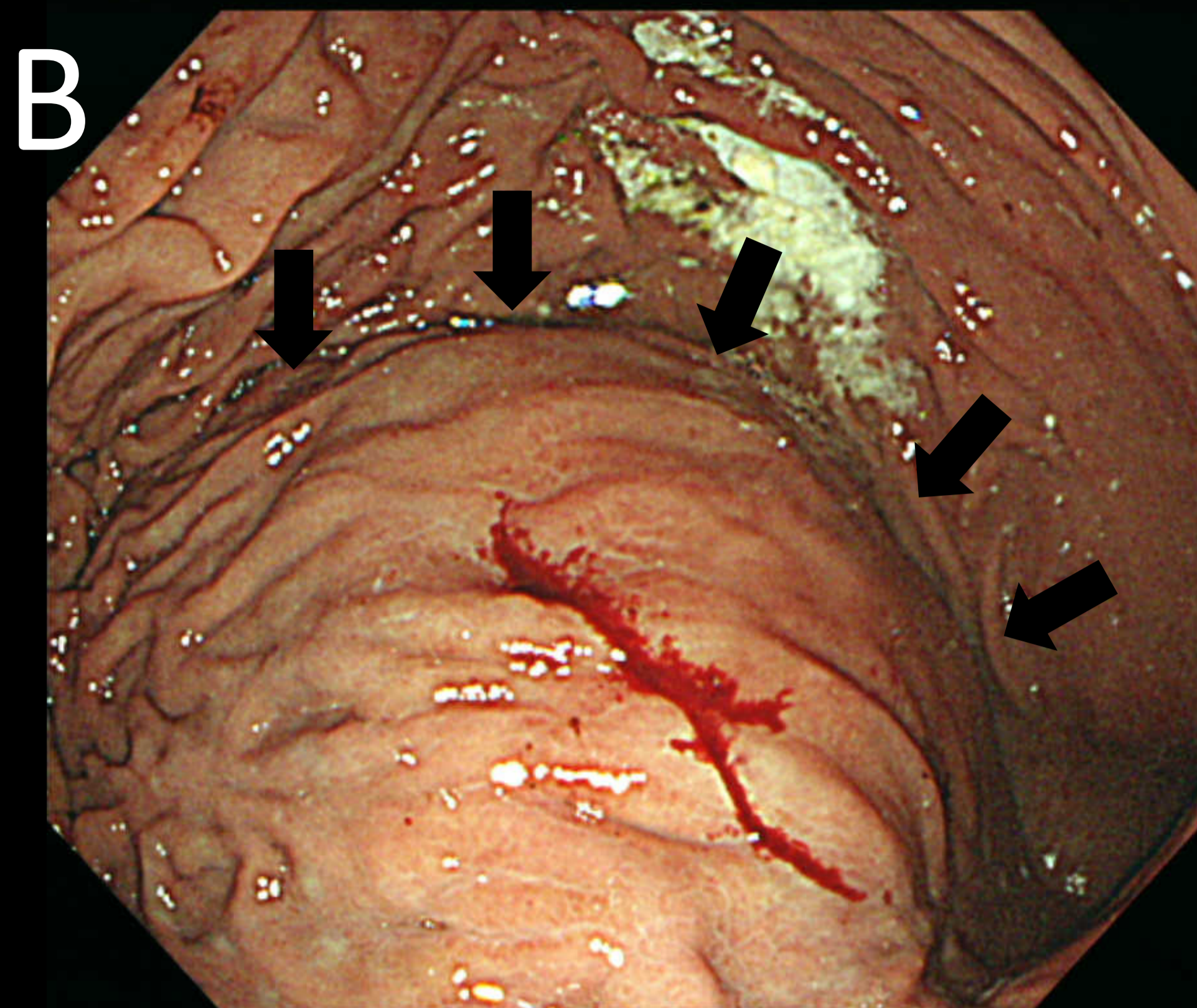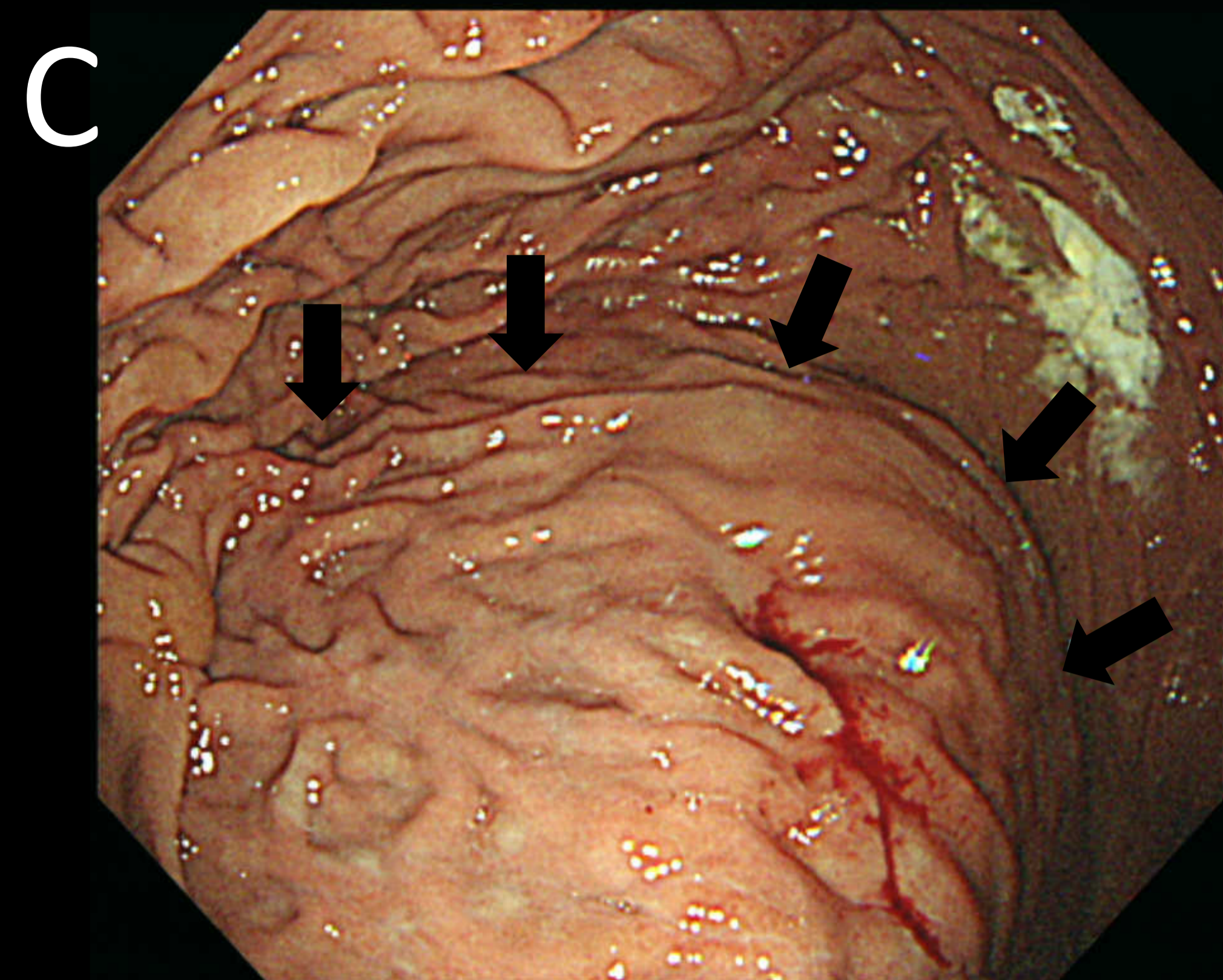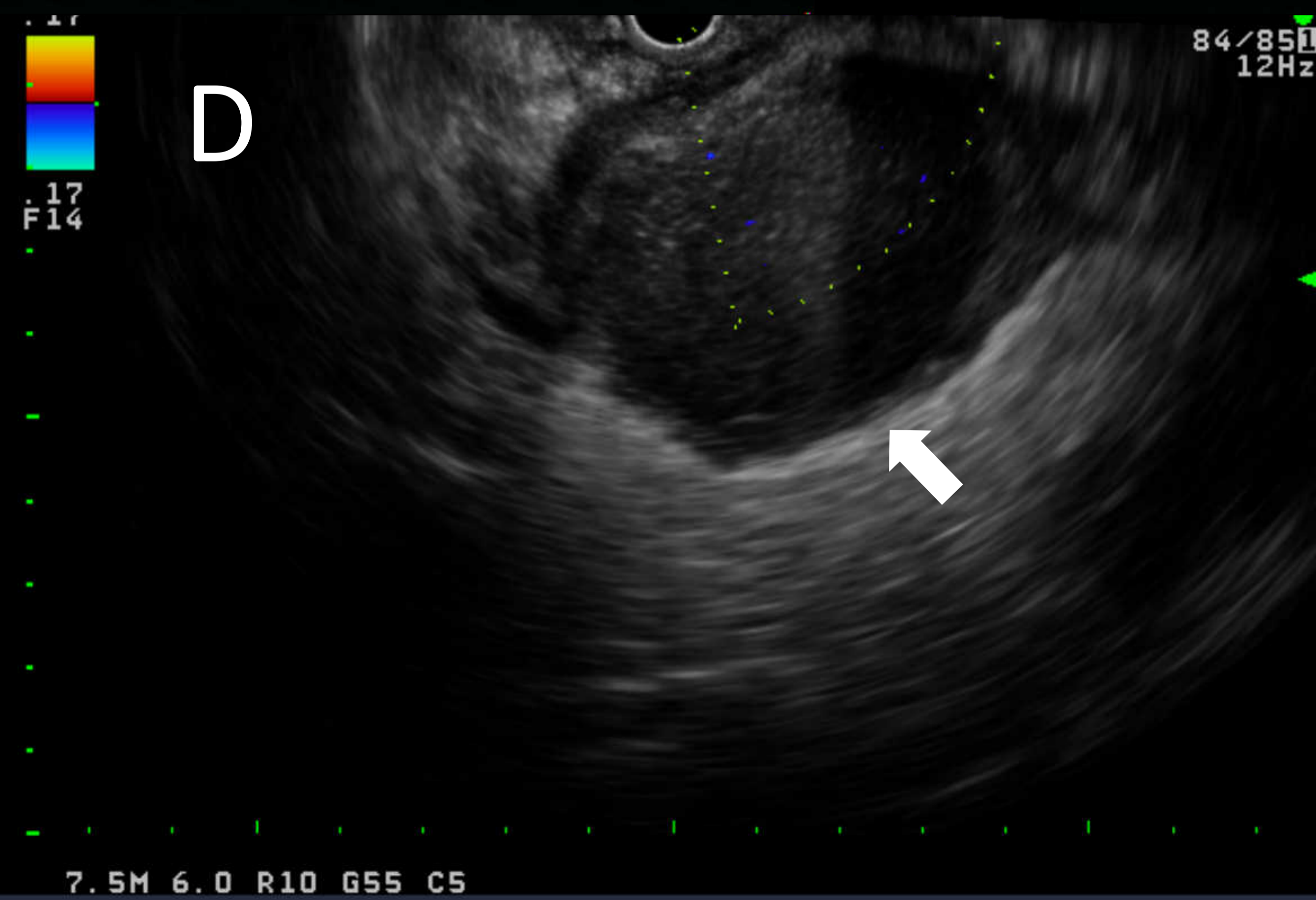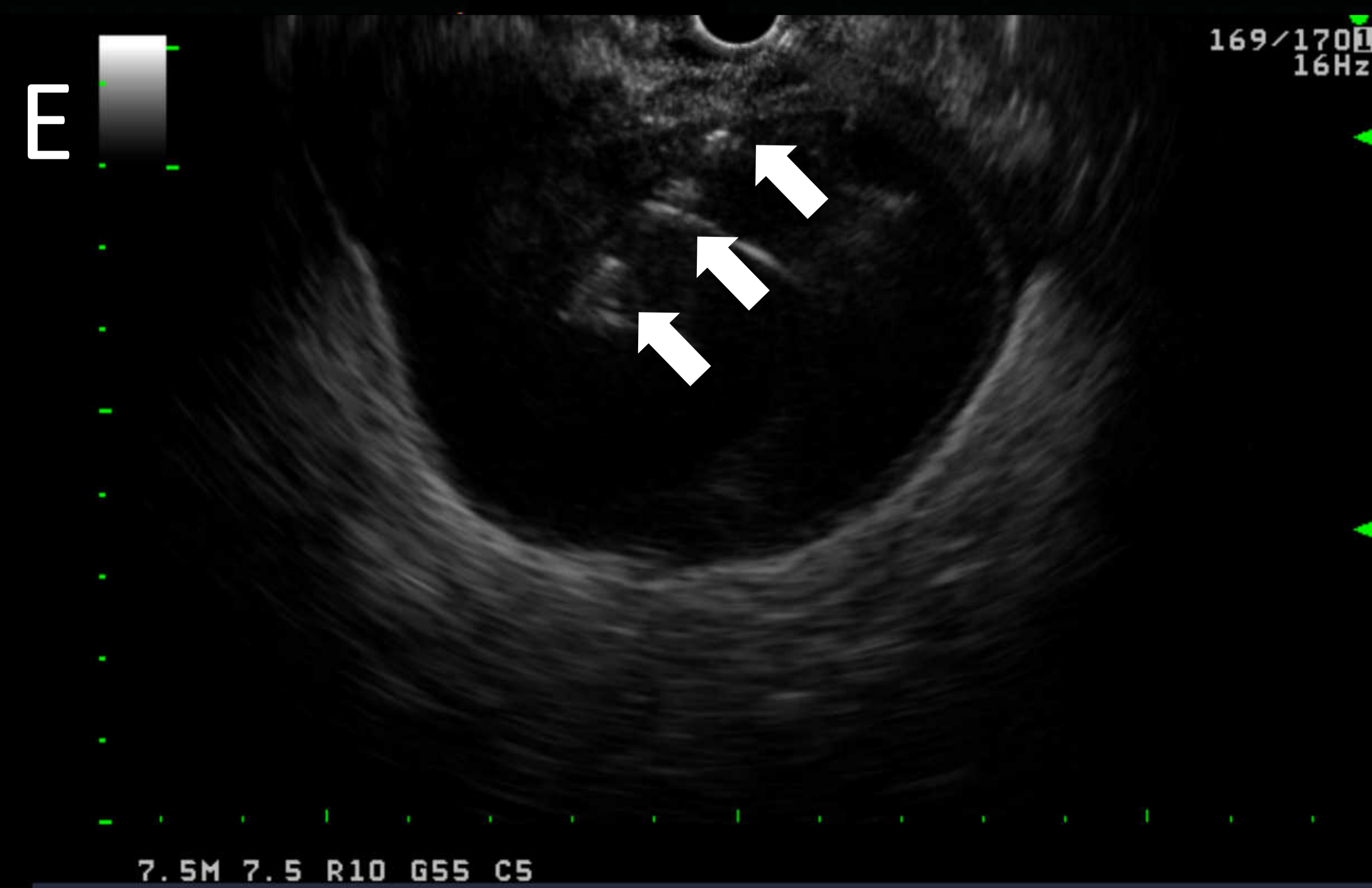

Supplement: Supplementary file 1 — Additional file 1. [file 12876_2020_1446_MOESM1_ESM.zip › Edwardsiella2R4.pdf]

A

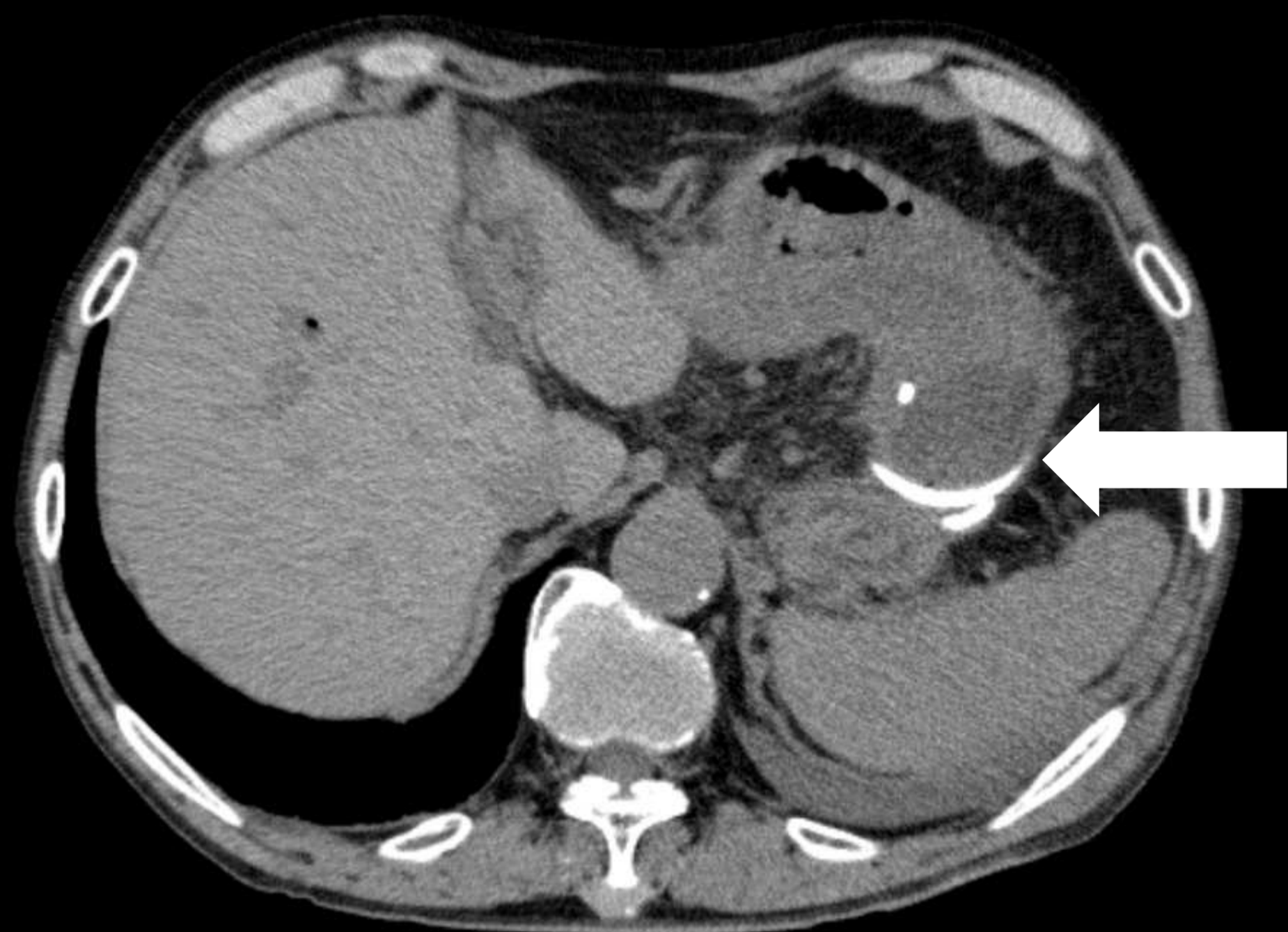

B

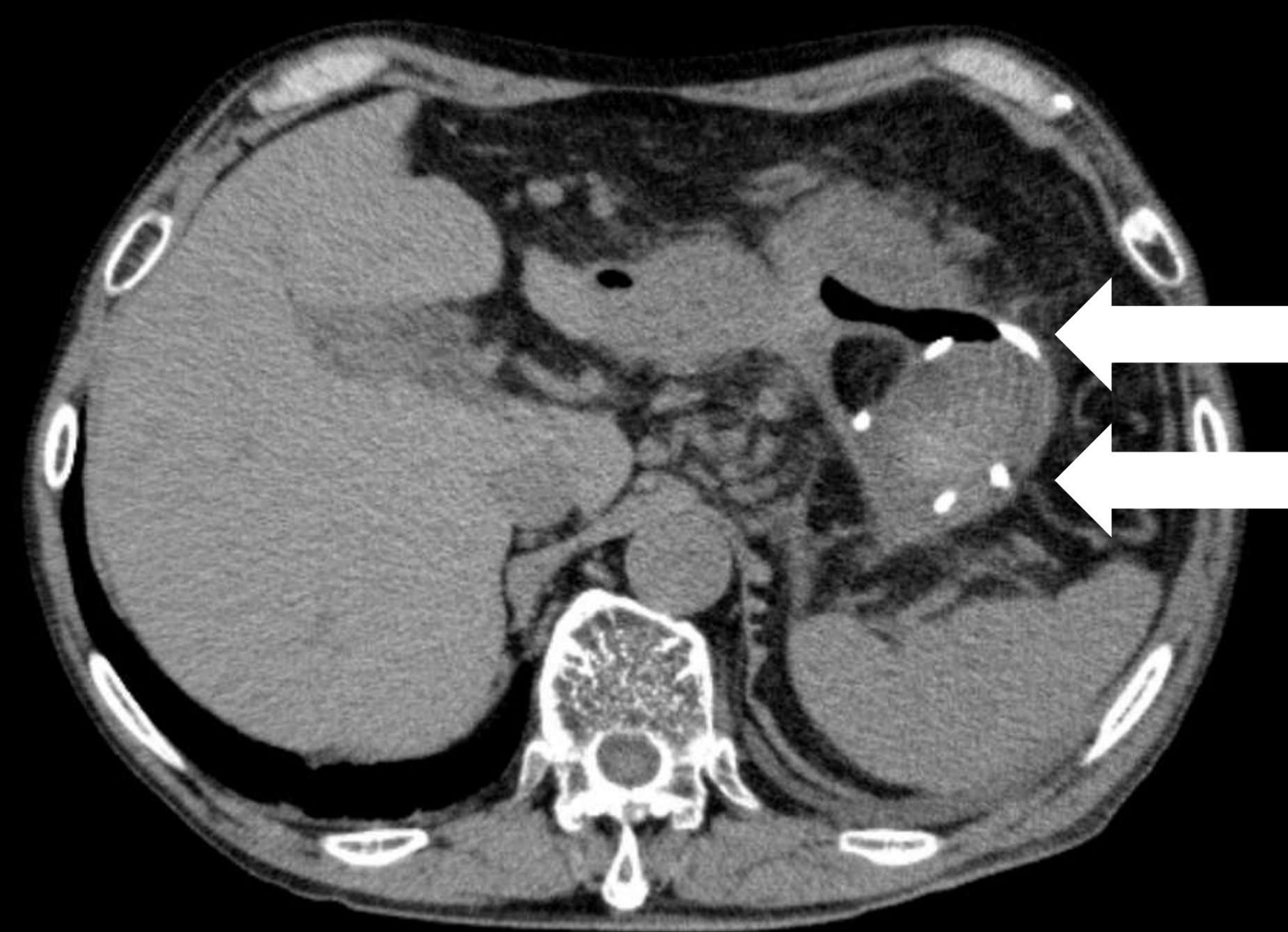

C

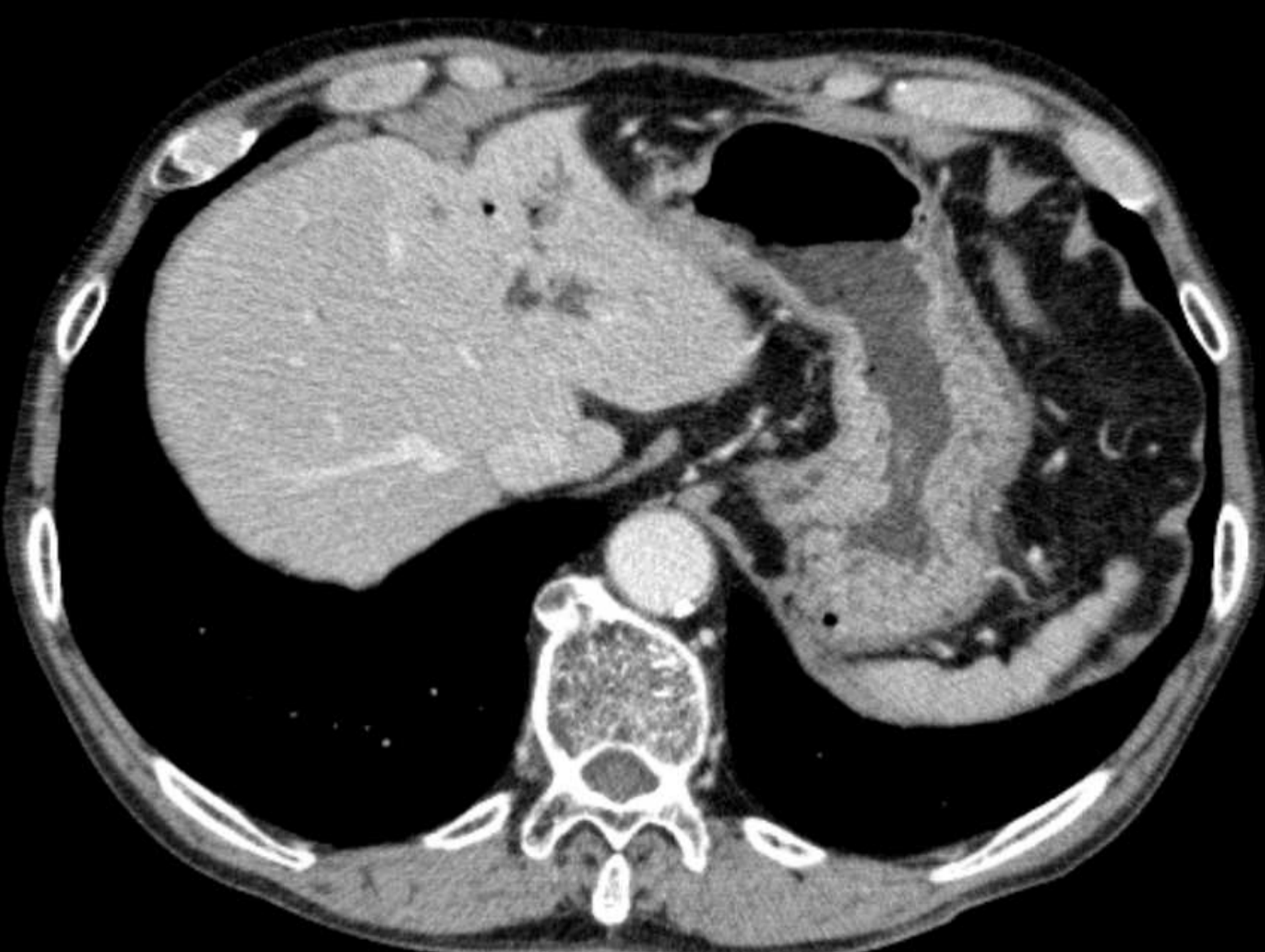

D

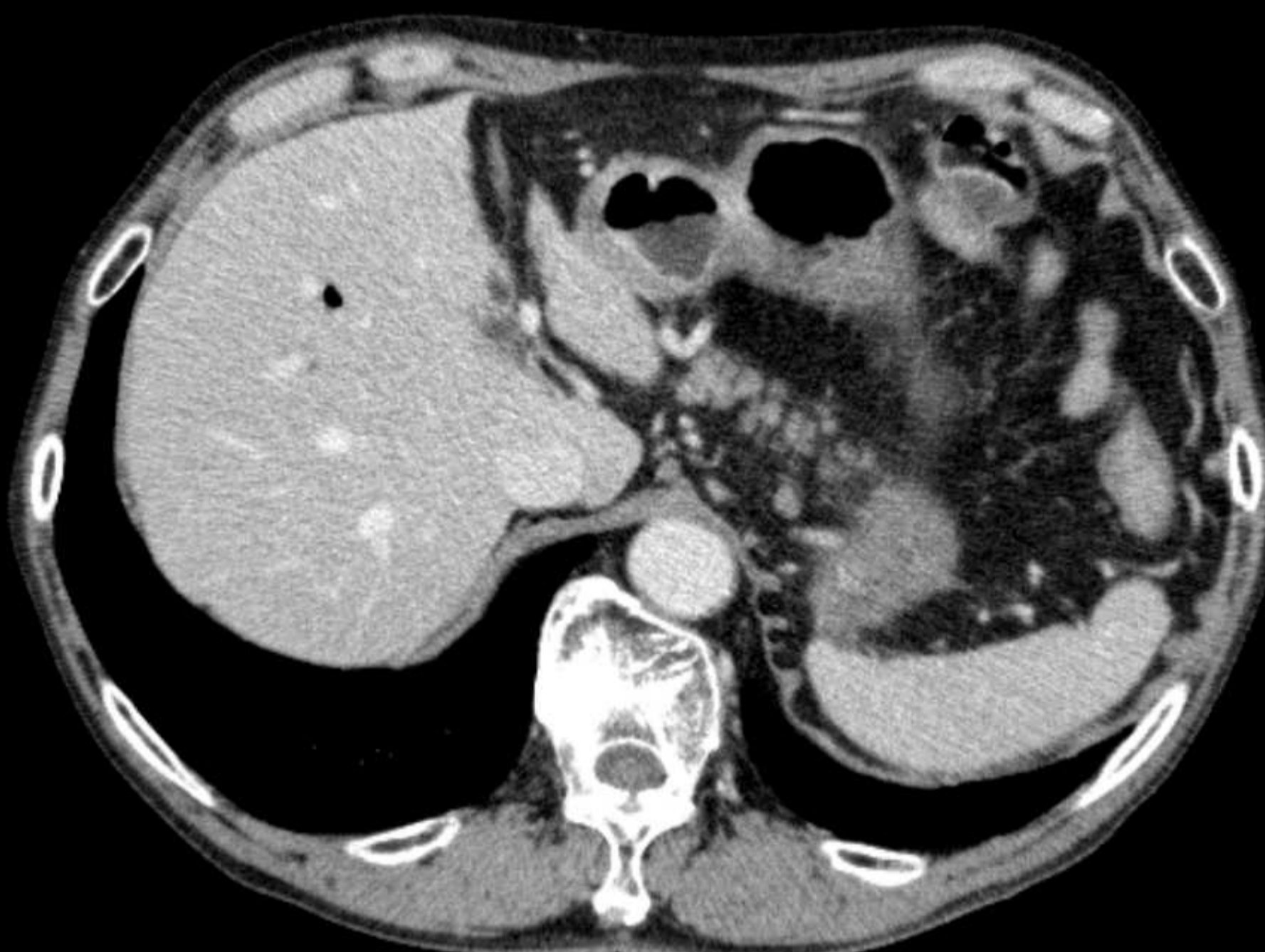

Supplement: Supplementary file 1 — Additional file 1. [file 12876_2020_1446_MOESM1_ESM.zip › Edwardsiella3R4.pdf]

A

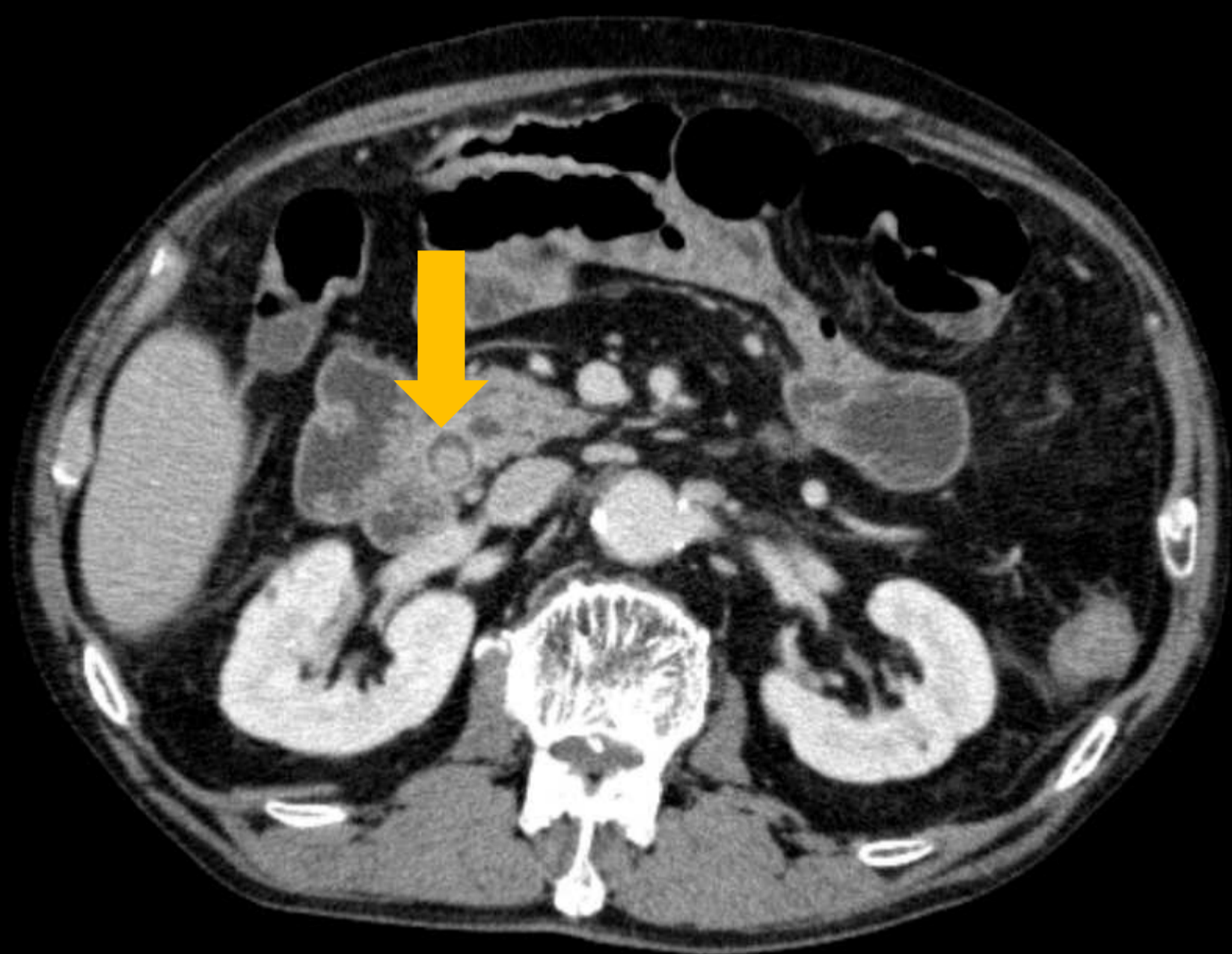

B

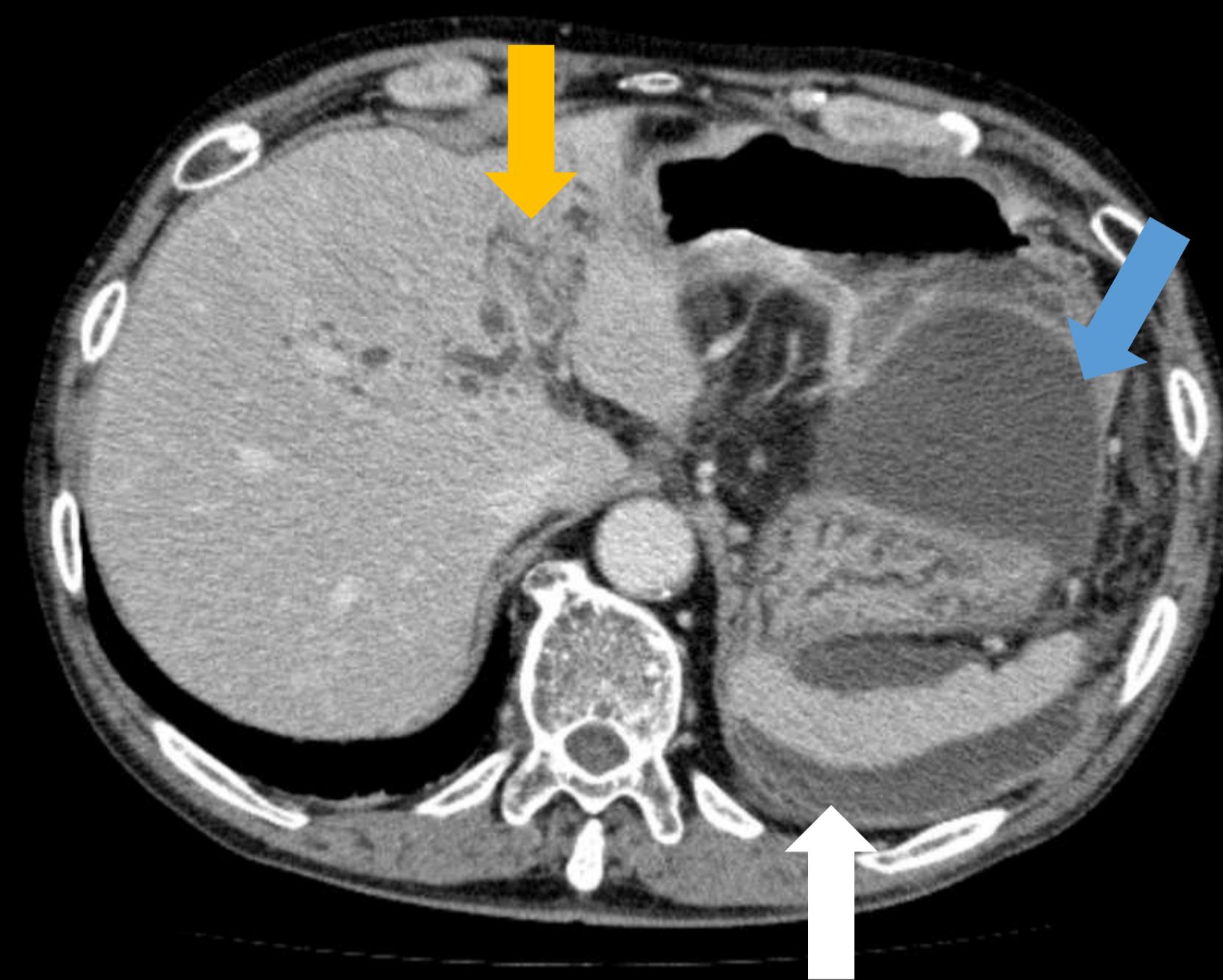

C

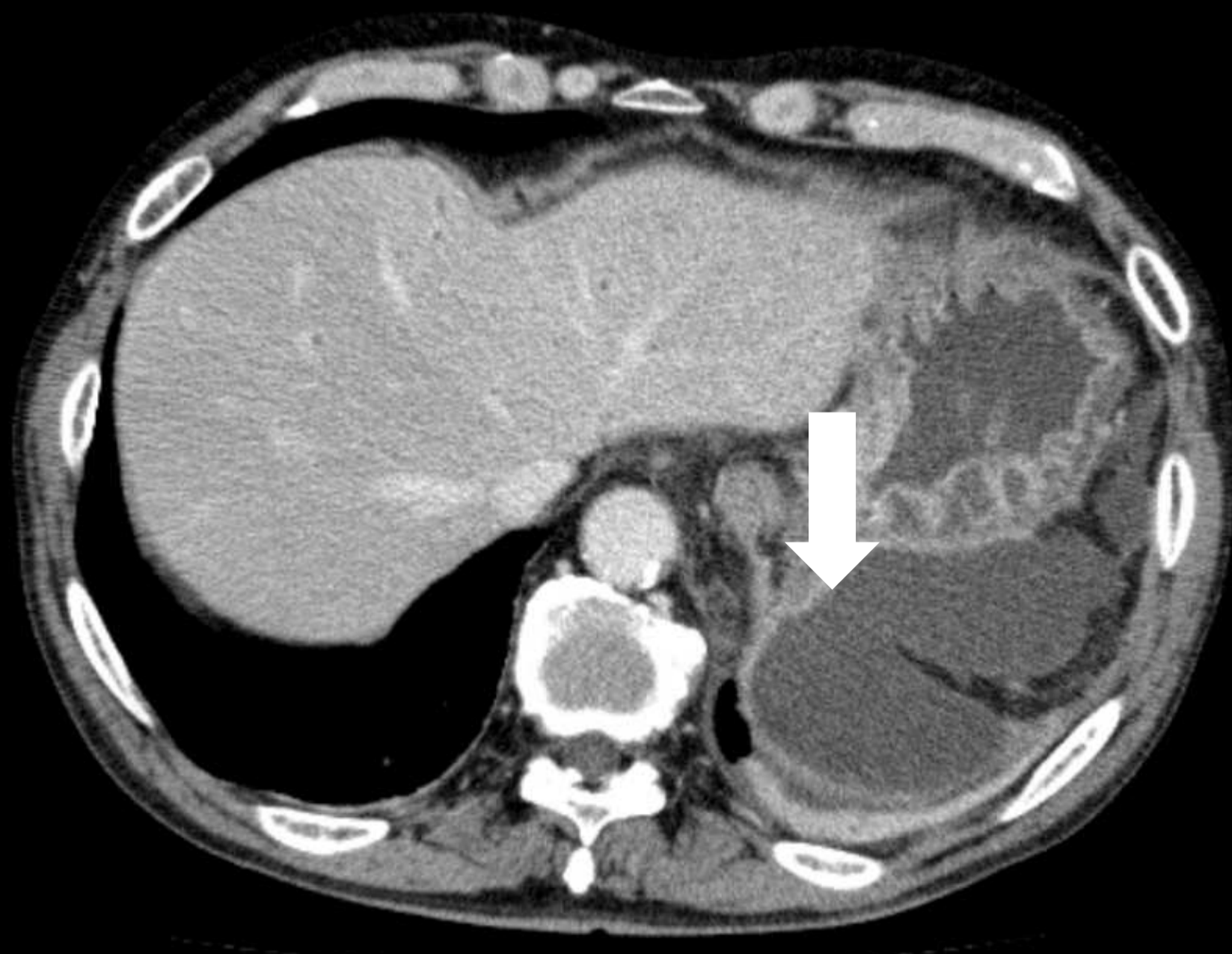

D

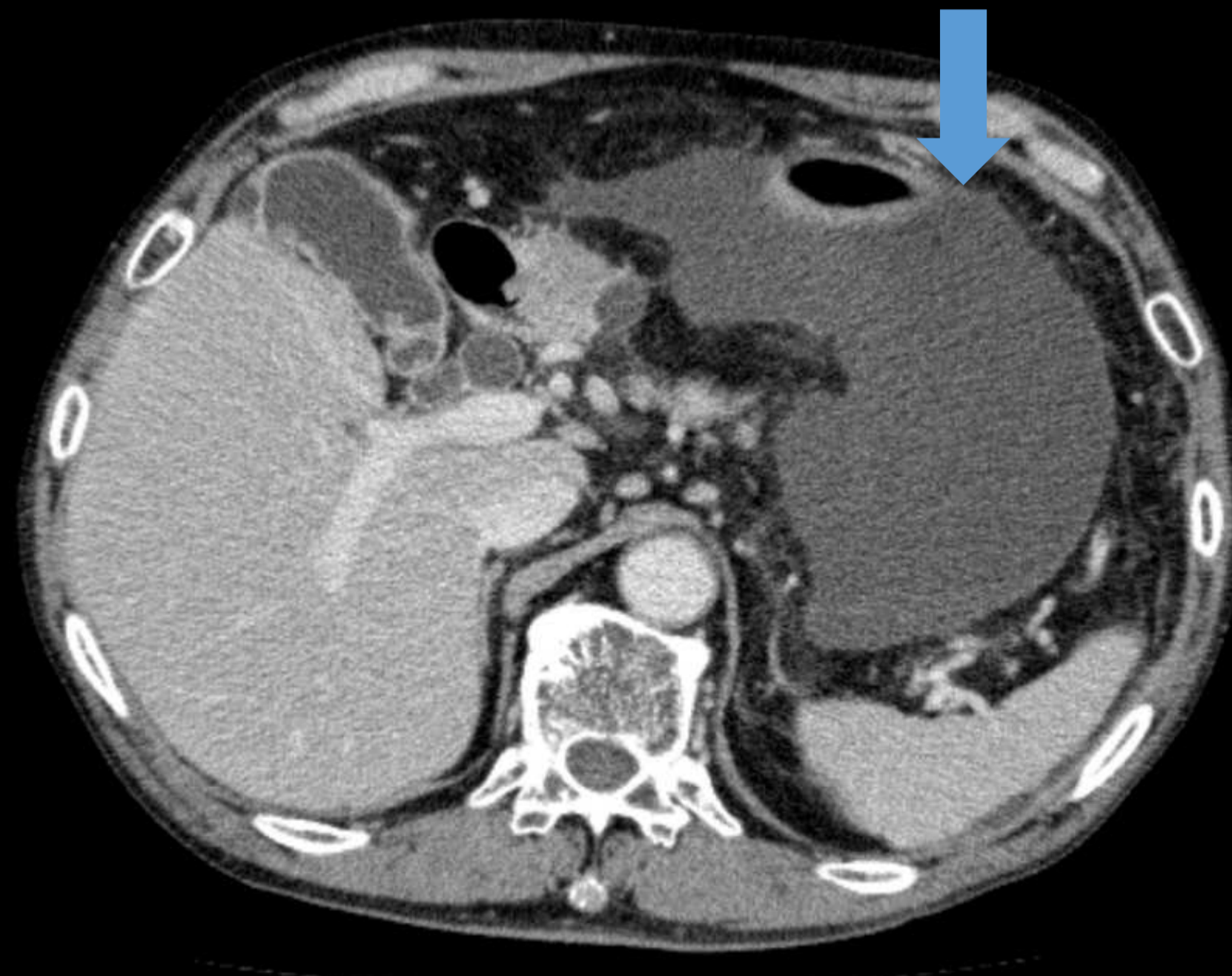

Supplement: Supplementary file 1 — Additional file 1. [file 12876_2020_1446_MOESM1_ESM.zip › EdwardsiellaR4.pdf]

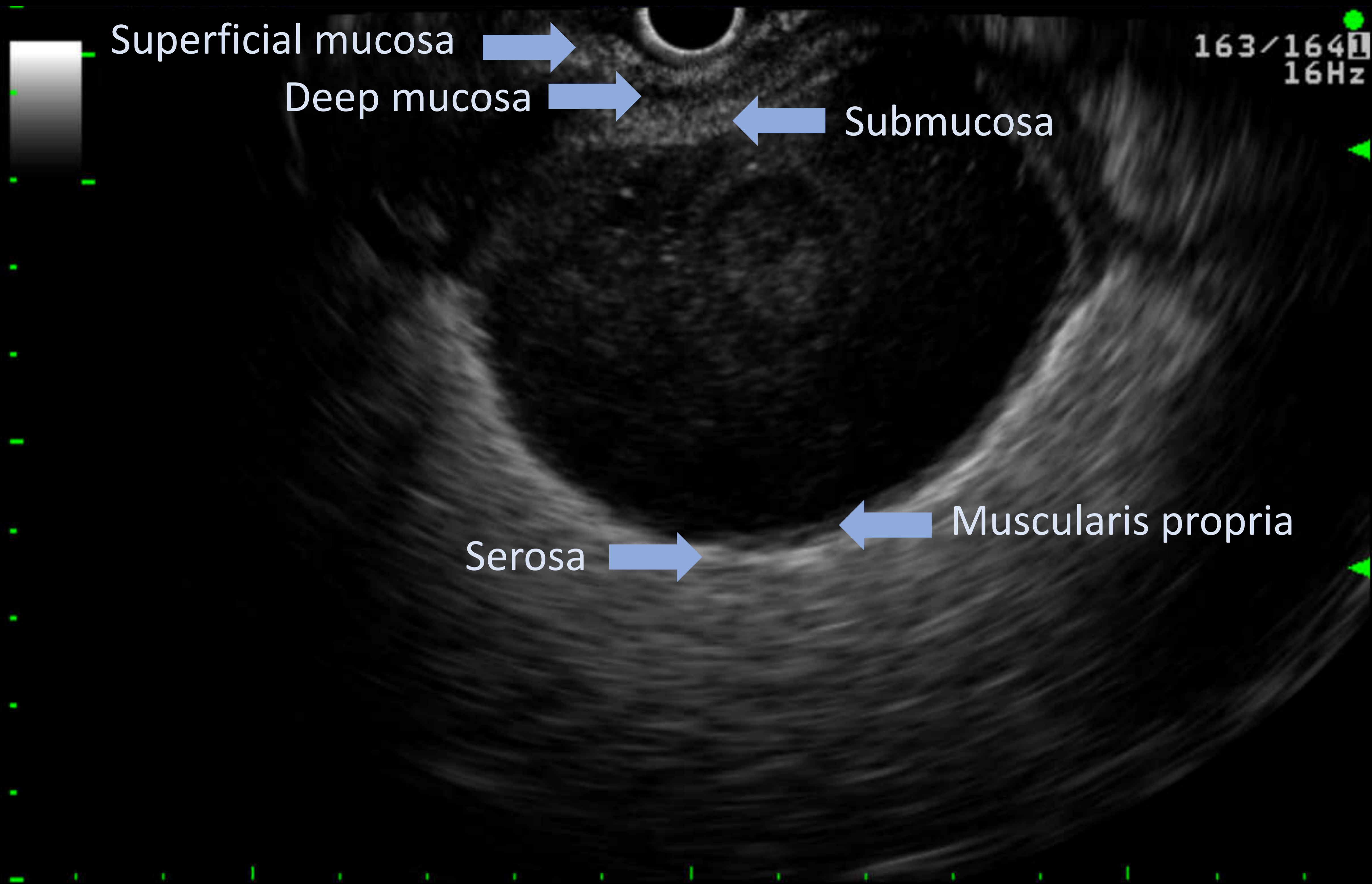

7.5M 7.5 R10 G55 C5

5:GF SCOPE

DVA: 100%

Supplement: Supplementary file 1 — Additional file 1. [file 12876_2020_1446_MOESM1_ESM.zip › EUS layerR4.pdf]
